# Wearable Sensor: An Emerging Data Collection Tool for Plant Phenotyping

**DOI:** 10.34133/plantphenomics.0051

**Published:** 2023-07-04

**Authors:** Cheng Zhang, Jingjing Kong, Daosheng Wu, Zhiyong Guan, Baoqing Ding, Fadi Chen

**Affiliations:** ^1^ College of Engineering, Nanjing Agricultural University, Nanjing 210095, China.; ^2^ State Key Laboratory of Crop Genetics & Germplasm Enhancement and Utilization, Key Laboratory of Biology of Ornamental Plants in East China, National Forestry and Grassland Administration, College of Horticulture, Nanjing Agricultural University, Nanjing 210095, China.; ^3^ Zhongshan Biological Breeding Laboratory, No.50 Zhongling Street, Nanjing 210014, China.

## Abstract

The advancement of plant phenomics by using optical imaging-based phenotyping techniques has markedly improved breeding and crop management. However, there remains a challenge in increasing the spatial resolution and accuracy due to their noncontact measurement mode. Wearable sensors, an emerging data collection tool, present a promising solution to address these challenges. By using a contact measurement mode, wearable sensors enable in-situ monitoring of plant phenotypes and their surrounding environments. Although a few pioneering works have been reported in monitoring plant growth and microclimate, the utilization of wearable sensors in plant phenotyping has yet reach its full potential. This review aims to systematically examine the progress of wearable sensors in monitoring plant phenotypes and the environment from an interdisciplinary perspective, including materials science, signal communication, manufacturing technology, and plant physiology. Additionally, this review discusses the challenges and future directions of wearable sensors in the field of plant phenotyping.

## Introduction

The global population is predicted to surge from its current 7.8 billion to 9.8 billion by 2050 [1,2], necessitating a marked increase in agricultural productivity to meet the growing demand for food. Plants, particularly crops, are crucial to agricultural production and provide the primary source of food for humans. However, crops are increasingly threatened by environmental and biological stresses that markedly affect their growth and yield [3]. Therefore, improving crop yields is essential to ensure food security. Plant phenomics has shown great potential in improving crop yields by systematically analyzing plant physiological characteristics, such as structure, color, form, size, texture, and other attributes, which are determined by both genotype (the internal factor) and environment (the external factor) [4]. Among them, the use of various data collection tools to acquire comprehensive phenotypes, also known as phenotyping, is essential [5–7].

Optical imaging is a commonly used data collection tool in phenotyping for acquiring information about plant phenotype states and dynamics [8], including physiology [9], temperature [10,11], and architecture [12]. Traditional optical imaging techniques rely on 2-dimensional methods, such as red-green-blue imaging [13,14], multispectral imaging [15,16], hyperspectral imaging [17], thermal imaging [18,19], and fluorescence imaging [20,21]. However, these techniques struggle to capture 3-dimensional (3D) plant structure information. To address this issue, 3D imaging techniques, such as passive stereo vision [22], passive structure from motion [23], active structured light [24], active time-of-flight camera [25], and active light detection and ranging (Lidar) [26,27], have been introduced to phenotyping. Despite their high throughput and temporal resolution, optical imaging-based phenotyping techniques face 2 major challenges. First, acquiring inner information, such as the inner structures in the plant canopy [28–31], is difficult because the light is often blocked by the outer layers. Second, measurements are susceptible to light conditions [8,32] and background reflectance [33] due to the reflection working mechanism of the techniques. Therefore, improving the spatial resolution and applicability of optical imaging-based phenotyping techniques in field environments is necessary.

Wearable sensors are an emerging data collection tool and a promising alternative to overcome the challenges of optical imaging in plant phenotyping. Originally designed for human health monitoring [34–36], wearable sensors are now being explored for plant research purposes [3,37]. Unlike the noncontact measurement of optical imaging, wearable sensors are directly attached to the epidermis of plants, enabling them to convert plant and environmental information into readable electrical signals in situ [38]. Due to their contact measurement mode, wearable sensors can detect not only inner plant information, including plant physiology underneath the epidermis [39–44], but also the microenvironment [45–47] that directly affects plant phenotypes. Additionally, wearable sensors are highly resistant to environmental interference, allowing them to offer high spatial resolution, convenience, and high accuracy in fields for plant phenotyping.

**Fig. 1.**
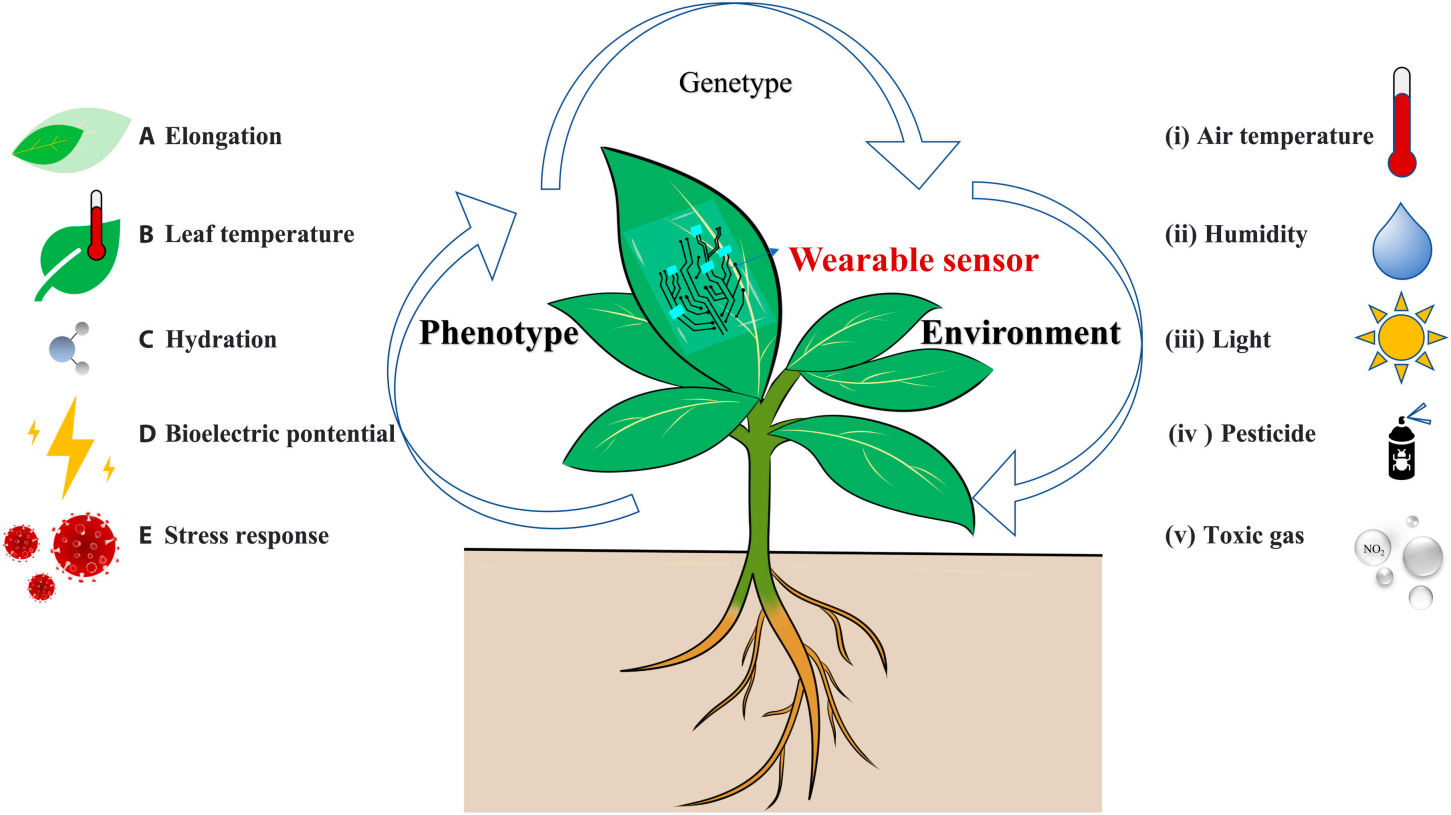
Wearable sensors for monitoring plant phenotypes and environment.

Despite their potential, wearable sensors have not fully realized their significance in plant phenotyping. The objective of this paper is to explore and expand the possibility of wearable sensors as an emerging data collection tool for plant phenotyping from an interdisciplinary perspective. The argument is supported by a comprehensive review of the progress of wearable sensors in monitoring plant phenotypes (including elongation, leaf temperature, hydration, bioelectric potential, and stress response) and environmental factors (including air temperature, humidity, light, pesticide, and toxic gas), as shown in Fig. 1. Additionally, the advantages of wearable sensors for collecting plant information, such as high temporal and spatial resolution [39], multifunctionality [45,47], and minimal invasiveness [37,48], are emphasized in practical applications. Lastly, the existing challenges of wearable sensors for plant phenotyping are outlined, and possible solutions are proposed.

## Wearable Sensors for the Measurement of Plant Phenotypes

Traditional plant phenotyping methods are constrained in spatial resolution and accuracy due to their noncontact measurement mode. However, the rapid development of wearable sensors, which features high spatial resolution, multifunctionality, and minimal invasiveness, provides a suitable tool for measuring plant phenotypes. In this section, we review the progress of wearable sensors in measuring plant phenotypes, such as elongation, leaf temperature, hydration, bioelectric potential, and stress response, as summarized in Table 1.

**Table 1.** Wearable sensors for the measurement of plant phenotypes.

Phenotypes	Sensor	Plant	Ref.
Elongation	A Ti/Au-based strain sensor	Barley and lucky bamboo	[50]
	A chitosan-based sensor	Cucumber	[54]
	A carbon nanotube/graphite-based strain sensor	*Cucurbita pepo*, *Solanum melongena* L.	[39]
	A liquid-alloy-based sensor	Sprout	[55]
Temperature	A tag sensor	/	[56]
	A “dust” network of wireless sensors	Melon	[40]
	An RFID-based system	Pumpkin	[58]
Hydration	A PI-based sensor	Tobacco	[64]
	A GO-based humidity sensor	*Epipremnum aureum*	[41]
	A graphene-based sensor	Maize	[69]
	A Cu-based flexible electronic sensor	Watermelon	[70]
Bioelectric potential	BDD electrodes	*Opuntia*	[73]
	BDD /Nafion and BDD/ Vylon electrodes	*Aloe* and *Opuntia*	[42]
	Thermogel-based morphable ionic electrodes	Sunflower and tobacco	[48]
	Self-adhering electrodes	*Dionaea muscipula*, *Arabidopsis thaliana*, and*Codariocalyx motorius*	[74]
Stress response	A graphene-based sensor array	Tomato leaf	[80]
	Conductive polymer electrodes	*Hosta* and pothos seedling	[82]
	Conductive polymer electrodes	Grape leaf	[44]

### Elongation

Elongation is an accurate indicator of plant growth [49], which aids in understanding the plant growth rhythm and response to environmental conditions [50]. The typical optical phenotyping method for measuring elongation is time-lapse imaging [51], which enables noninvasive and continuous monitoring. However, this method has limitations, as the optical path can be easily blocked by other growing branches or leaves. However, wearable sensors distributed on the surface of plants allow for in-situ monitoring of tensile strain, which can be converted to plant elongation. Nevertheless, the contact measurement mode requires wearable sensors to have sufficient stretchability to adapt to the continuous growth of plant organs, so that they will not break or restrict the growth of plants.

**Fig. 2.**
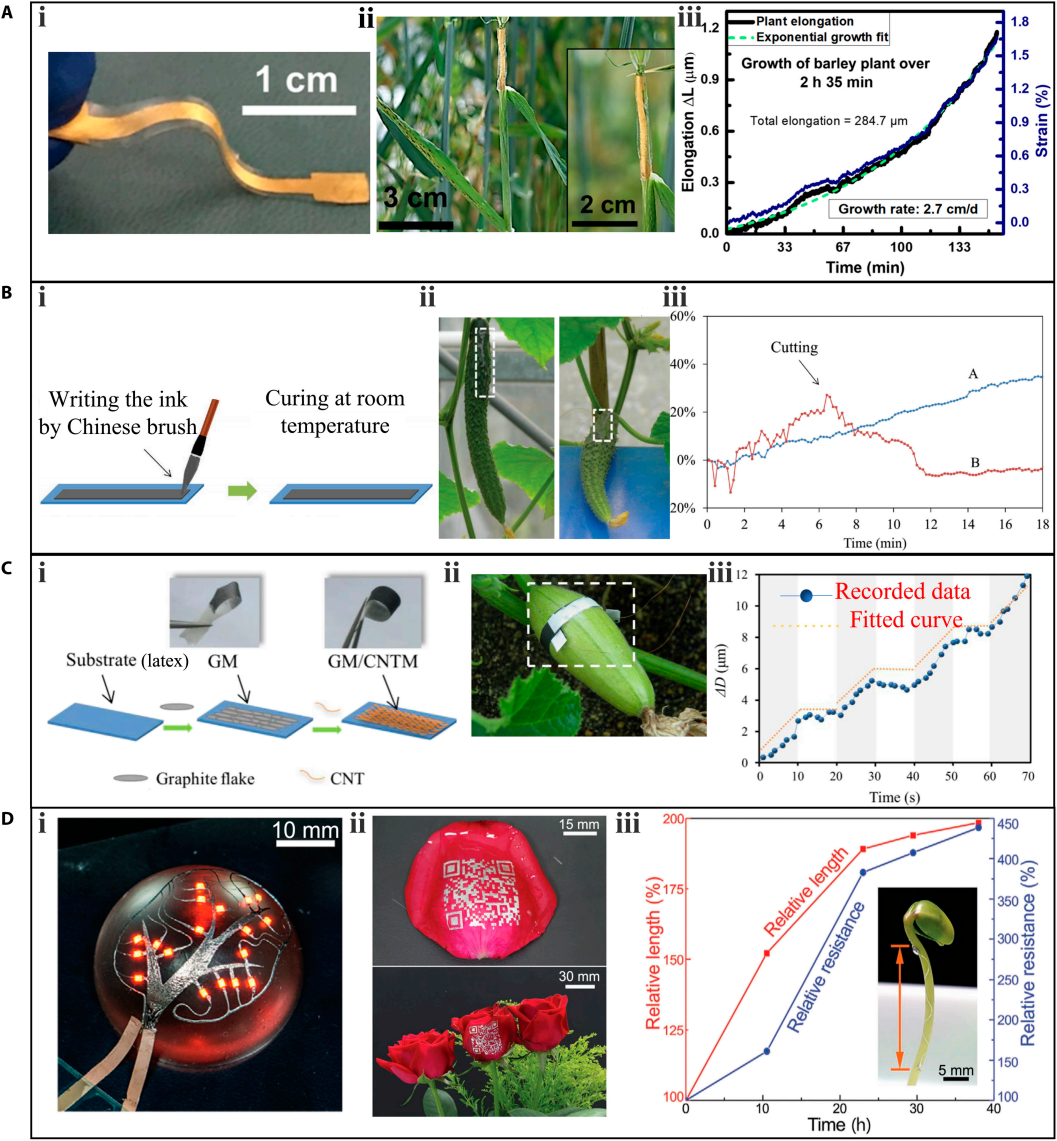
Wearable sensors for measuring plant elongation. (A) A Ti/Au-based stretchable strain sensor: (i) a digital photo of the strain sensor, (ii) a photograph of the sensor system attached on a barley plant, and (iii) a plot displaying the real-time measurement of the leaf elongation on barley plant. Adapted under the terms of the CC-BY 4.0 Creative Commons Attribution License. [50] Copyright 2018, the Authors, published by Springer Nature. (B) A chitosan-based direct writing flexible sensor: (i) schematic illustration of the fabrication, (ii) the sensors printed on 2 cucumber fruits (one remained on the stem, and the other was disconnected from the stem) used for the measurement, and (iii) real-time monitoring of the cucumber growth. Adapted with permission. [54] Copyright 2017, Wiley. (C) A graphite/carbon nanotube-based strain sensor: (i) key steps in fabricating the graphite membrane (GM)/carbon nanotube membrane (CNTM) strain sensor, (ii) the sensor mounted on a *Cucurbita pepo* plant, and (iii) stepwise growth recorded by the wearable sensor. Adapted with permission. [39] Copyright 2019, Elsevier. (D) A liquid-alloy-based sensor: (i) a fractal circuit printed on the 3D surface of a silicone semisphere, (ii) LA codes printed on roses, and (iii) the relative length of the sprout and the relative resistance of the sensor as a function of growth time. Reproduced with permission. [55] Copyright 2020, Wiley.

To achieve high stretchability, materials and manufacturing techniques are critical. Nassar et al. [50] proposed a stretchable strain sensor that uses flexible, stretchable, and biocompatible materials to monitor plant elongation (Fig. 2Ai). In their study, a thin Ti/Au metal film was deposited on a stretchable substrate polydimethylsiloxane (PDMS) as a strain sensing material. To eliminate the influence of moisture on resistance, the sensor was encapsulated by another hydrophobic PDMS layer. Notably, the researchers also implemented a buckling technique in which the PDMS layer was prestrained to improve the stretchability of the sensor to 35%. Finally, the sensor showed a linear detection range of 0% to 22% strain, corresponding to an elongation range of 0 to 3.75 mm. The gauge factor of the sensor was 3.9, which is sufficient to monitor the micrometer elongations of plant growth. The strain sensor was anchored on barley stem to measure growth (Fig. 2Aii), and the response of the sensor to plant growth was plotted in Fig. 2Aiii. In the growth period of 2 h and 35 min, the total strain detected was 1.6%, which corresponded to a leaf elongation of 284.7 μm.

Another approach to improving stretchability is embedding conductive materials into elastic polymer composites [52,53]. Tang et al. [54] presented a direct-written flexible sensor (Fig. 2Bi) by mixing graphite powder and chitosan solution in a certain proportion. The resulting stretchable flexible sensor could be directly brushed onto the desired position. To prevent interference from humidity, the sensor was sealed with rubber pieces. Experimental results showed that the sensor could reach a maximum strain of 60%. The sensors were directly written on 2 cucumber fruits as groups A and B (Fig. 2Bii) to monitor their elongation. As shown in Fig. 2Biii, the resistance of the sensor in group A continuously increased as the fruit grew. In group B, the resistance of the sensor first increased but then decreased. This transition occurred when the entire fruit was disconnected from the stem, indicating that the fruit stopped growing and started shrinking after being cut.

Latex, a type of stretchable polymer, can provide excellent stretchability for wearable sensors in plant phenotyping [39]. As shown in Fig. 2Ci, a stretchable latex substrate was coated with graphite ink and carbon nanotube ink to enhance the sensor's stretchability and gauge factor to 150% and 352, respectively. The resulting sensor was mounted on a *Cucurbita pepo* fruit for circumferential elongation monitoring (Fig. 2Cii). The high sensitivity and temporal resolution of the sensor enabled it to discover an interesting phenomenon: the growth of the *Cucurbita pepo* follows a rhythmic pattern. As shown in Fig. 2 Ciii, the diameter of the pepo increased by 12 μm in 70 s, with a growth period of 10 s and a stagnating period of 10 s, alternately. This strain sensor demonstrated the capability of dynamically measuring elongation at the micrometer scale.

Gallium-based liquid alloy (LA) featured with high fluidity and electrical conductivity was employed to fabricate a strain sensor with a high stretchability of 200% [55]. The LA circuit is also self-morphing, allowing it to adapt to the irregular shapes of substrates. For instance, a fractal circuit was printed on the 3D surface of a silicone semisphere (Fig. 2Di). This feature enables the LA-based sensor to have a robust interface with plants. The sensor was printed directly onto the plant epidermis, such as rose and bean sprout (Fig. 2 Dii to iii). The sensor was able to detect more than 200% elongation of the bean sprout (Fig. 2Diii).

### Leaf temperature

There are marked differences between plant leaf temperature and air temperature. Monitoring the leaf temperature and analyzing the temperature difference between the leaf and the air can help determine whether plants are under water stress [56]. Unlike traditional infrared thermal imaging methods [57], wearable sensors are minimally affected by environmental factors. In leaf temperature measurement research, much effort has been focused on the data transmission of wearable sensors.

**Fig. 3.**
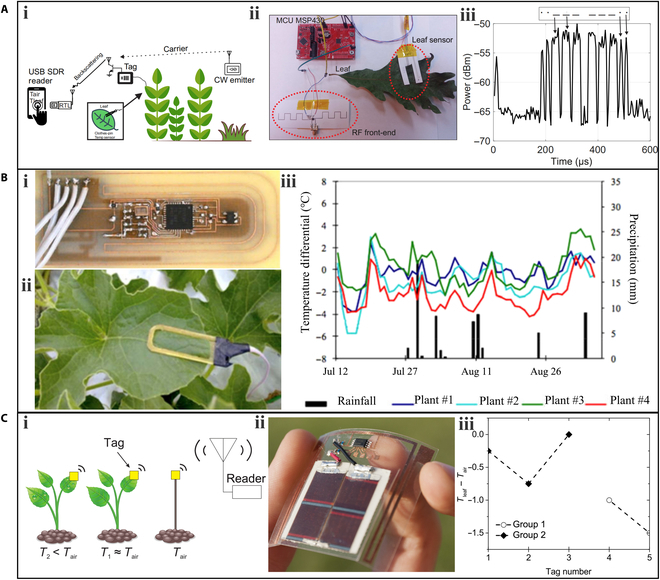
Wearable sensors for the measurement of plant leaf temperature. (A) A tag-sensor node based on backscattering communication: (i) a schematic of backscatter communication, (ii) the sensor node and tag design, and (iii) the backscatter signal and power level in the time domain. The transmitted Morse symbols “.. − − − − − − ..” corresponded to 28 °C. Reproduced with permission. [56] Copyright 2017, IEEE. (B) A wireless temperature sensor network based on ZigBee: (i) the top view of the temperature sensor prototype, (ii) a temperature sensor mounted on a melon leaf, and (iii) temperature differentials recorded by the sensors and their relationship with precipitation. Reproduced with permission. [40] Copyright 2017, Cambridge University Press. (C) An accurate leaf-compatible temperature sensing system based on RFID technology: (i) a schematic of the sensing system, (ii) a photo of the transponder on polylactic acid, and (iii) the leaf-to-air temperature difference of the 2 experiment groups. Reproduced with permission. [58] Copyright 2019, IEEE. USB, universal serial bus; CW, continuous wave; MCU, microcontroller; RF, radio frequency.

Wireless communication is widely used in agricultural applications due to its convenience and low cost. Daskalakis et al. [56] proposed a tag-sensor node for leaf temperature measurement based on the wireless backscattering principle, which transmits data through an incident radio-frequency signal without requiring a battery or power source (Fig. 3Ai). The sensors were fabricated using low-cost inkjet-printing technology with nanoparticle inks and silver epoxy. The study employed a "clothes-pin" scheme, placing 2 sensors on the top and back of a leaf, respectively, to measure air temperature and leaf temperature (Fig. 3Aii). The communication part of the sensor exploited backscatter Morse code modulation on an 868-MHz carrier emitter signal. A Morse code symbol corresponded to a value of air temperature. For example, the Morse symbol “.. − − − − − − ..” in Fig. 3Aiii corresponded to 28 °C.

Palazzari et al. [40] proposed a wireless temperature sensor network that uses a 2.4-GHz ZigBee protocol for data transmission. The researchers designed a clip-shaped temperature sensor (Fig. 3Bi) that can be easily fixed to the edge of a leaf. Four sensors were clipped to 4 leaves of a melon crop in Umbria, Italy, for in-field measurements of the temperature differential between the leaf and air (Fig. 3Bii). They found that the temperature differentials recorded by the 4 sensors displayed similar trends and were influenced by rainfall (Fig. 3Biii), which is associated with the water stress condition of the plant.

Another wireless technology, radio-frequency identification (RFID), which is characterized by low installation and maintenance costs, has also received increasing attention. Palazzi et al. [58] proposed an accurate autonomous leaf-compatible temperature sensing system composed of an EM4325 sensor chip and an RFID transponder (Fig. 3Ci). To design lightweight and flexible circuits, a 0.29-mm-thick polylactic acid layer was used as the substrate. The complete RFID transponder weighed less than 3 g (Fig. 3 Cii). Temperature sensor nodes were placed on the surface of pumpkin leaves to detect the temperature difference between the leaf and air (Fig. 3Ciii). The pumpkins in group 1 were hydrated as they were watered after the sensors were placed, while the pumpkins in group 2 were under drought stress as they were not watered. The average temperature difference between the leaves and air in group 1 was −1.25 °C, while that in group 2 was about −0.33 °C, indicating that water stress level is negatively correlated with the leaf temperature difference.

### Hydration

Water content and water movement are crucial factors in plant growth. In addition to measuring leaf temperature to indirectly reflect whether plants are subjected to water stress, direct measurement of plant hydration is another option. Traditional phenotyping methods for monitoring plant water content include thermal imaging [59] and terahertz imaging [60], which require laboratory settings [61]. Wearable sensors offer a solution for in-field measurement of plant hydration, but the interface between the sensor and the plant must be robust to accurately acquire hydration information.

One strategy is to use a clamp. In 2012, Atherton et al. [62] proposed a microfabricated thermal sensor device with a thin-film microheater for analyzing the moisture content of leaves by monitoring thermal resistance. The sensor was fixed to the leaf using a plastic clamp. Oren et al. [63] also used the clamp strategy, proposing a multiplex graphene oxide (GO)-based relative humidity (RH) sensor to track water transport inside maize plants. The sensor was adhered to the bottom of a 1-mm-deep chamber in acrylic glass, which was fixed onto the leaf's surface using lightweight plastic clamping slabs and screws. The disadvantage of this clamp strategy is the relatively complicated installation process, and the mechanical compressive force that may damage the clamped plant organs.

**Fig. 4.**
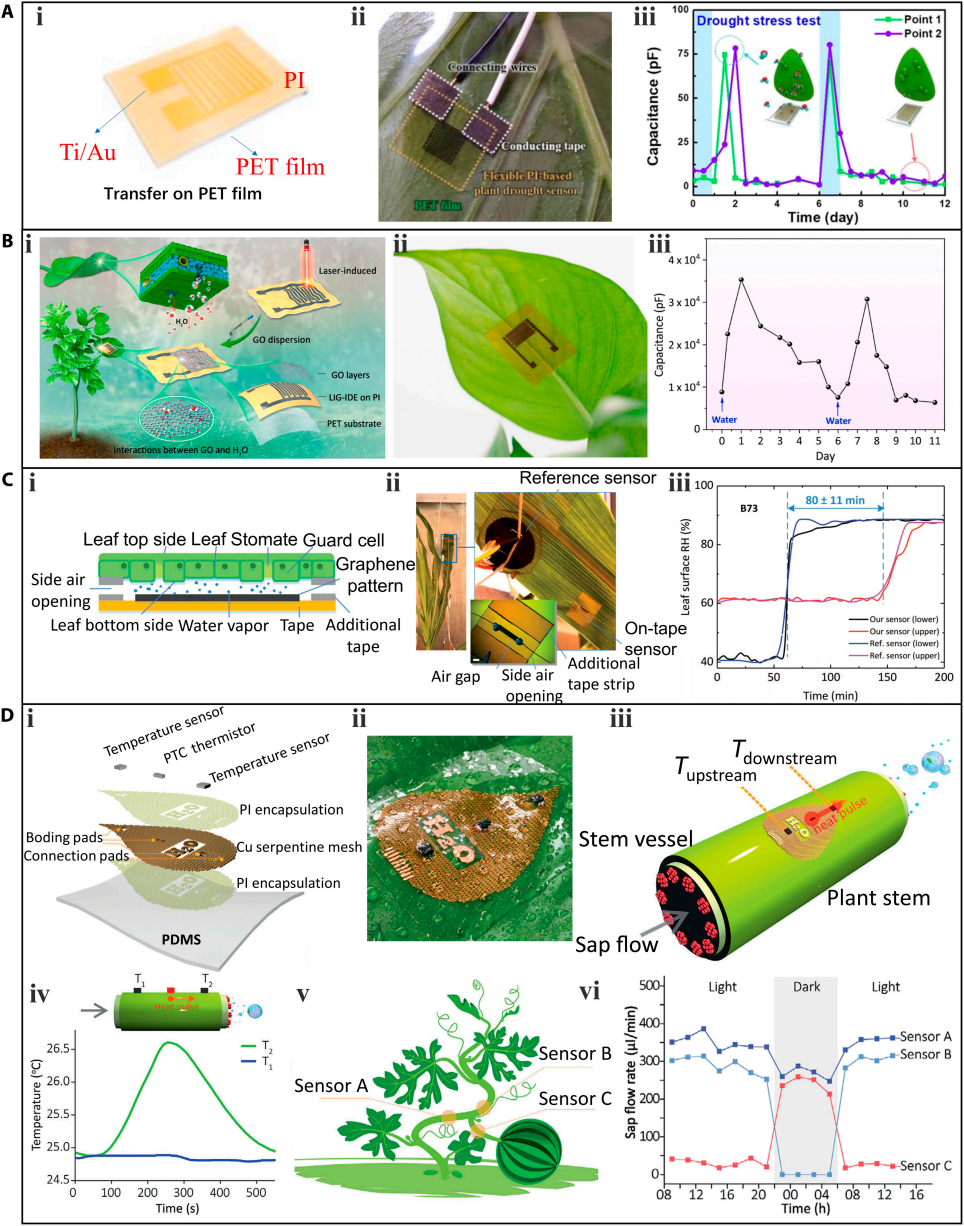
Wearable sensors for the measurement of plant hydration. (A) A PI-based plant drought sensor: (i) a structure diagram of plant drought sensor based on flexible PI, (ii) an optical image of the plant drought sensor mounted on the lower surface of the leaf, and (iii) the response of *Nicotiana tabacum* to drought stress. Reproduced under the terms of the CC-BY 4.0 Creative Commons Attribution License. [64] Copyright 2018, the Authors, published by MDPI. (B) A GO-based humidity sensor: (i) the manufacturing process of flexible humidity sensor, (ii) a photo of the GO-based humidity sensor mounted on the lower surface of the leaf of *Epipremnum aureum*, and (iii) the monitoring of capacitance response of the humidity sensor to drought stress. Reproduced with permission. [41] Copyright 2020, Elsevier. (C) A graphene-based moisture sensor: (i) a schematic diagram of the sensor placement and the mechanism of detection, (ii) a photograph of the graphene sensor and a commercial reference sensor mounted on the back of a maize leaf, and (iii) real-time monitoring of the RH level at the maize of inbred line B73 after plant irrigation. Reproduced with permission. [69] Copyright 2017, Wiley. (D) A flexible electronic sensory system to track plant sap flow: (i) an exploded view illustration of the plant-wearable sensor, (ii) an optical image of the sensor installed on a pathos leaf, (iii) a schematic diagram of the detection principle, (iv) temperatures measured with a sap flow from left to right, (v) schematic of the 3 sensors arrangement on a watermelon plant, and (vi) variation of the sap flow rates measured by the 3 sensors. Reproduced under the terms of the CC-BY 4.0 Creative Commons Attribution License. [70] Copyright 2021, the Authors, published by Wiley. PET, polyethylene terephthalate; PTC, positive temperature coefficient.

A more convenient and plant-friendly strategy is to use adhesive tape that is nontoxic, although this approach is only viable for wearable sensors with high flexibility. Otherwise, they cannot intimately fit the plant epidermis. Many efforts have been devoted to fabricating flexible hydration sensors. Im et al. [64] developed a plant drought sensor based on a polyimide (PI) film to monitor the moisture status of tobacco plants. The plant drought sensor was formed by depositing Ti/Au electrodes onto a flexible PI film, which acted as both the sensing element and supporting substrate. The sensor was then peeled from the glass and transferred to a 1-side sticky polyethylene terephthalate film with high flexibility, which facilitated its installation on the plant. Figure 4Ai shows the structure of the plant drought sensor, while Fig. 4 Aii shows the sensor attached to the lower surface of a *Nicotiana tabacum* leaf. The moisture released by the transpiration of the leaf increased the capacitance of the PI film, and monitoring the capacitance could therefore deduce the hydration status of the plant. Figure 4Aiii shows the response of the plant drought sensor capacitance over time during a measurement period, where watering occurred every 6 d, and the capacitance value rapidly increased after each watering.

To increase the sensitivity of wearable sensors for monitoring plant water status, Lan et al. [41] proposed a humidity sensor based on GO coated on a PI film. The manufacturing process (Fig. 4Bi) of the sensor involved the in-situ synthesis of a laser-induced graphene (LIG) interdigital electrode (IDE) on a flexible PI film using laser direct writing technology [65–68]. Then, a thin and uniform GO film was drop-casted onto the LIG-IDE to serve as the sensing element. The sensor was installed on the backside of the leaf of *Epipremnum aureum* (Fig. 4Bii), and the capacitance changes of the leaf surface over time were shown in Fig. 4Biii. Similarly, the capacitance of GO increased after watering, indicating the sensor's capability of monitoring plant water status and transpiration in real time with high sensitivity.

Besides capacitance-type moisture sensors, resistance-type moisture sensors have also been applied in plant hydration monitoring. Oren et al. [69] proposed a graphene-based moisture sensor using a high-resolution graphene patterning and transferring method. The desired graphene pattern was created within the prepatterned negative features on the surface of the PDMS substrate and transferred onto the target substrate using a unique "Drop cast-Dry-Stick-Peel" (D2SP) method. The resistance of the graphene would increase with moisture. The schematic illustration of sensor placement and detection mechanism is shown in Fig. 4Ci. Two fabricated moisture sensors and 2 commercial reference sensors were installed on leaves of the lower and upper sections of a maize plant (inbred line B73) to monitor the moisture of leaves (Fig. 4Cii). Figure 4Ciii displays the monitoring results. The resistances of the sensors attached on the lower and upper sections started to increase 55 and 135 min after irrigation, respectively. This result indicates that it takes about 80 min for water to move inside the maize from lower to upper sections.

In addition to monitoring leaf moisture, wearable sensors have also been developed to monitor sap flow in plant stems. Chai et al. [70] introduced a flexible electronic sensory system that can continuously track plant sap flow. The schematic diagram of the sensory system is shown in Fig. 4Di, which consisted of 2 temperature sensors and 1 positive temperature coefficient thermistor enclosed between 2 PI layers on a flexible PDMS substrate. This sensory system is highly flexible and can be conformably attached to plants (Fig. 4Dii). The sensing mechanism of the sensor is illustrated in Fig. 4Diii. After attaching the sensory system to a plant stem, the thermistor generates heat, which is transported in the direction of the flow, resulting in temperature anisotropy. As the flow is from left to right, the downstream sensor (T_2_) exhibits a temperature increase with time and then returns to the original temperature (Fig. 4Div). Conversely, when the flow direction is opposite, the temperature of T_1_ increases. Since the stem flow is the medium of the heat transfer, the difference in temperature (Δ*T*) between the 2 sensors can be converted to flow rate. To investigate the internal water distribution of watermelon, 3 sensors were mounted on a basal stem and 2 adjacent branches near a watermelon fruit. As shown in Fig. 4Dv, sensor A detected the flow in the basal stem of the watermelon while sensors B and C monitored the flow in the leaf and fruit branches, respectively. The investigation result is illustrated in Fig. 4 Dvi. During the light period, the sap flow rate of the leaf branch detected by sensor B was markedly higher than that of sensor C, and the result was reversed during the dark period. This indicates that most of the water in the basal stem was allocated to the leaf branch during the light period due to leaf photosynthesis and transpiration, while during the dark period, the basal water was mostly allocated to the watermelon fruit due to the cessation of leaf photosynthesis and respiration.

### Bioelectric potentials

Bioelectric potentials are vital for regulating life activities in plants and can change rapidly in response to external stimuli [71]. The conventional method of measuring bioelectric potentials involves inserting hard electrodes into tissues [72], which can cause damage to plants. The use of flexible electrode sensors as a minimally invasive phenotyping tool allows for direct attachment to the plant's surface to measure bioelectric potentials, causing minimal damage to the plant and enabling continuous measurement.

**Fig. 5.**
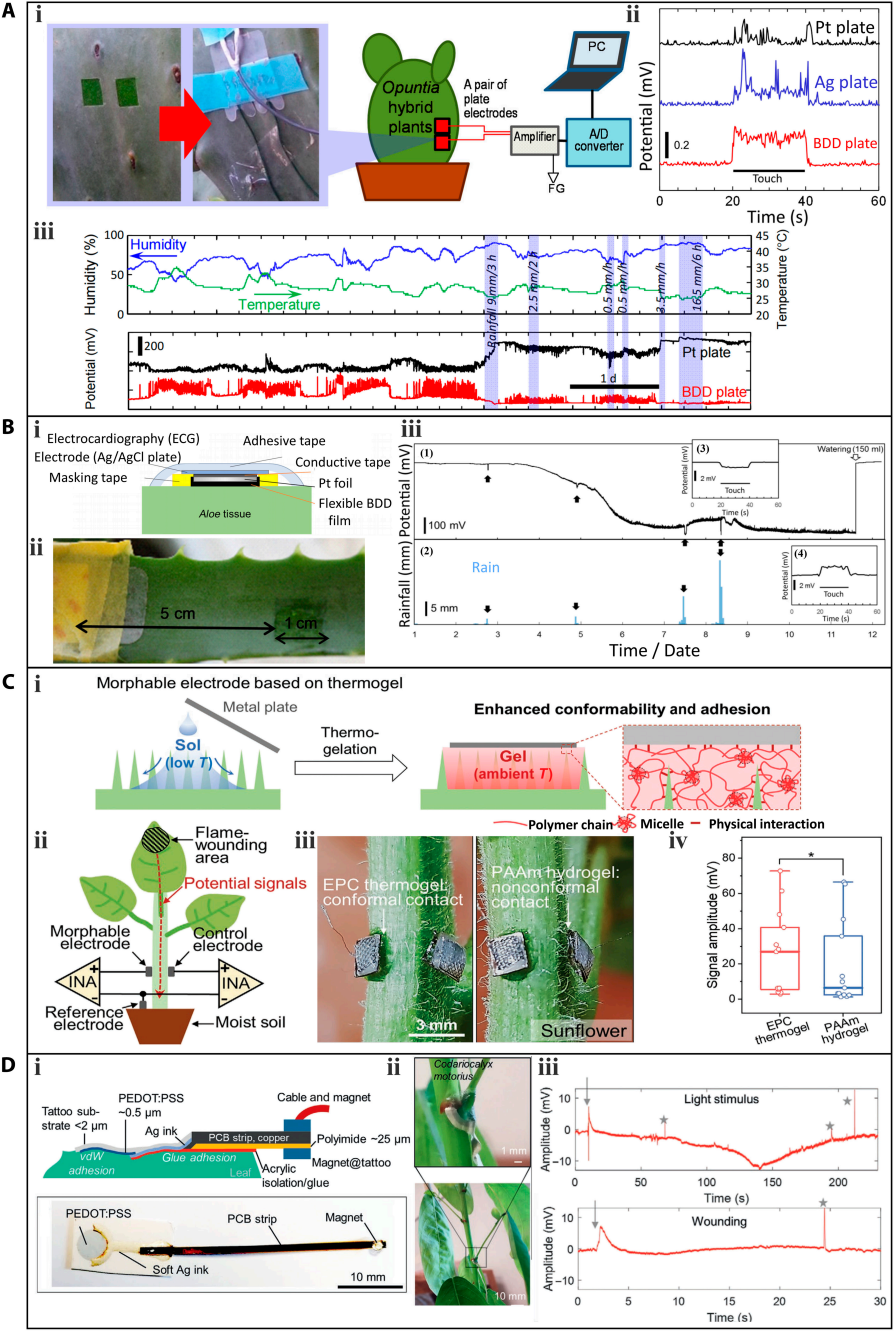
Wearable sensors for the measurement of plant bioelectric potentials. (A) A sensitive plant monitoring system with BDD electrodes: (i) a photo and schematic of experimental setup for detecting bioelectric potentials of potted *Opuntia* hybrid plants, (ii) bioelectric potentials in potted *Opuntia* hybrid plants caused by a finger touch, and (iii) bioelectric potential changes caused by environment variation recorded by the BDD and Pt electrodes. Reproduced under the terms of the CC-BY 4.0 Creative Commons Attribution License. [73] Copyright 2015, the Authors, published by MDPI. (B) Flexible BDD electrodes: (i) schematic illustration of a film-type electrode mounted on an *Aloe* leaf, (ii) BDD sensor elements and masking tape, and (iii) (1) potential signals recorded by the flexible BDD electrodes over 11 d, (2) rainfall over 11 d, (3) response to the finger touch stimulus, and (4) response after 150-ml watering. Adapted under the terms of the CC-BY 4.0 Creative Commons Attribution License. [42] Copyright 2017, the Authors, published by MDPI. (C) Thermogel-based morphable ionic electrodes: (i) schematic illustration of the adhesion process of morphable electrode onto hairy plants, (ii) measurement of potential changes induced by flame wounding, (iii) photographs showing plate electrodes adhered on a hairy sunflower stem through the thermogel and PAAm hydrogel, and (iv) potential signals read from the thermogel and PAAm hydrogel on sunflower stems. Reproduced with permission. [48] Copyright 2021, Wiley. (D) Self-adhering surface electrodes: (i) an illustration and image of the self-adhering electrodes, (ii) a photo of the electrodes applied on the pulvinus of *Codariocalyx motorius* leaves, and (iii) recorded bioelectrical signals with light and wounding stimuli. Reproduced under the terms of the CC-BY 4.0 Creative Commons Attribution License. [74] Copyright 2021, the Authors, published by Wiley. FG, frame background; INA, instrumentation amplifier; EPC, the thermogelling polymer consisted of hydrophilic poly(ethylene glycol) (PEG), thermoresponsive poly(propylene glycol) (PPG), and hydrophobic biodegradable polycaprolactone (PCL) segments, named poly(PEG/PPG/PCL urethane) and denoted as EPC; PCB, printed circuit board.

Analogous to the measurement of plant hydration, in order to accurately monitor the bioelectric potentials, it is necessary to ensure that the flexible electrode is tightly integrated with the leaf. However, different plants have varying epidermal structures according to plant physiology, making surface attachment of the flexible electrode and plant different. For plants with smooth skins such as *Opuntia* and *Aloe*, Ochiai et al. [73] attached a boron-doped diamond (BDD) electrode sensor to a piece of green phloem tissue to monitor bioelectric potentials (Fig. 5Ai). Metal electrode (Pt and Ag) sensors were also characterized for comparison. The BDD sensor could detect obvious changes in bioelectric potentials when a finger touched the hybrid surface of *Opuntia* (Fig. 5Aii) or when environmental factors such as temperature and humidity changed. The measurement could be continued for 7 d, indicating the long-term monitoring capability of the BDD sensor. Although the sensitivity of the BDD sensor was 5 to 10 times higher than that of the metal sensors, the signal stability was unsatisfactory.

To improve the stability of the bioelectric signal, a composite-type sensor was developed by incorporating BDD powder and resin (BDD/Nafion and BDD/Vylon) [42]. The sensor was attached to the surface of an *Aloe* leaf (Fig. 5Bi), and covered with masking tape to prevent electromagnetic noise interference, though it may interfere with plant physiological behaviors by hindering light incidence and gas exchange (Fig. 5Bii). The sensor successfully recorded bioelectric potential changes induced by rainfall or finger touch (Fig. 5Biii). The upper part (1) of Fig. 5Biii shows the changes in bioelectric potential of the *Aloe* over a 12-d period, and the lower part (2) shows the rainfall log. The bioelectric potential changed at the rainfall points indicated by the black arrows. *Aloe* returned to its original bioelectric potential after pouring enough water at Day 11. Moreover, the BDD/resin composite sensor illustrated a higher signal stability demonstrated higher signal stability compared to the pure BDD sensor (Fig. 5Aiii).

However, for plants with hairy surfaces such as sunflowers and tobacco, traditional electrodes may not fit well on the hair-like surfaces, which can hinder detection of the bioelectric potential. To address this issue, a morphable ionic electrode based on a thermogel was used [48]. As shown in Fig. 5Ci, the thermogel was in a sol state at low temperatures, allowing it to easily penetrate the villi on hair-like surfaces and provide a large adhesive area. Then, its temperature gradually increased to room temperature and induced transformation from the sol state to a viscoelastic gel state. The gel state offered a higher mechanical strength than the sol state, forming a strong adhesion between the plant's hairy surface and the electrode sensor. It is worth noting that the environmental temperature should not decrease to a temperature lower than the transition temperature, or the sensor will lose the adhesion to the plant and terminate the measurement.

The thermogel morphable electrode was used to detect potential changes induced by flame wounding on a sunflower stem, and a control electrode made of the hydrogel polyacrylamide (PAAm) was also attached for comparison (Fig. 5Cii). The thermogel showed good adhesion to the stem, while the PAAm hydrogel barely adhered (Fig. 5Ciii). Both electrodes detected bioelectrical potential changes within the plant stem induced by flame wounding of a leaf. However, the signal intensity recorded by the thermogel electrode was markedly higher than that recorded by the PAAm hydrogel electrode (Fig. 5Civ), indicating that the morphable electrode based on the thermogel is capable of recording clearer and more stable bioelectric signals from hairy plants.

Self-adhering surface electrodes that rely on van der Waals forces have been reported for measuring bioelectric potentials in plants [74]. As shown in Fig. 5Di, these devices are fabricated by patterning poly(3,4-ethylenedioxythiophene) polystyrene sulfonate (PEDOT:PSS) pads (a conductive polymer) and Ag ink (a stretchable silver conductor paste) on a tattoo transfer paper. When the PEDOT:PSS layer touches the leaf, the electrode self-adheres onto the plant surface via van der Waals adhesion. These self-adhering electrodes are suitable for recording bioelectric potentials in plants executing fast movements. As shown in Fig. 5Dii, the electrode was attached to the pulvini of *Codariocalyx motorius* leaves, which were exposed to light stimuli or partially cut. The electrodes were able to record a time-dependent electrical response (Fig. 5Diii), with the gray arrows representing the time point of the stimulus and the gray stars indicating the corresponding potential response. These experimental results demonstrate the potential application of self-adhering electrodes in measuring the bioelectrical potentials generated by different stimuli in plants.

### Stress response

Plants are frequently exposed to biotic or abiotic stresses, such as pathogen infections [75], ultraviolet [76], and ground-level ozone [77], which can hinder plant growth and alter some physiological characteristics. It is crucial to measure the plant's stress response at an early stage and take timely intervention. Traditional phenotyping methods for measuring stress response are based on visual identification [78], but these methods may not detect early-stage stress responses. Wearable sensors offer a potential solution to this problem, enabling real-time monitoring and prompt intervention.

**Fig. 6.**
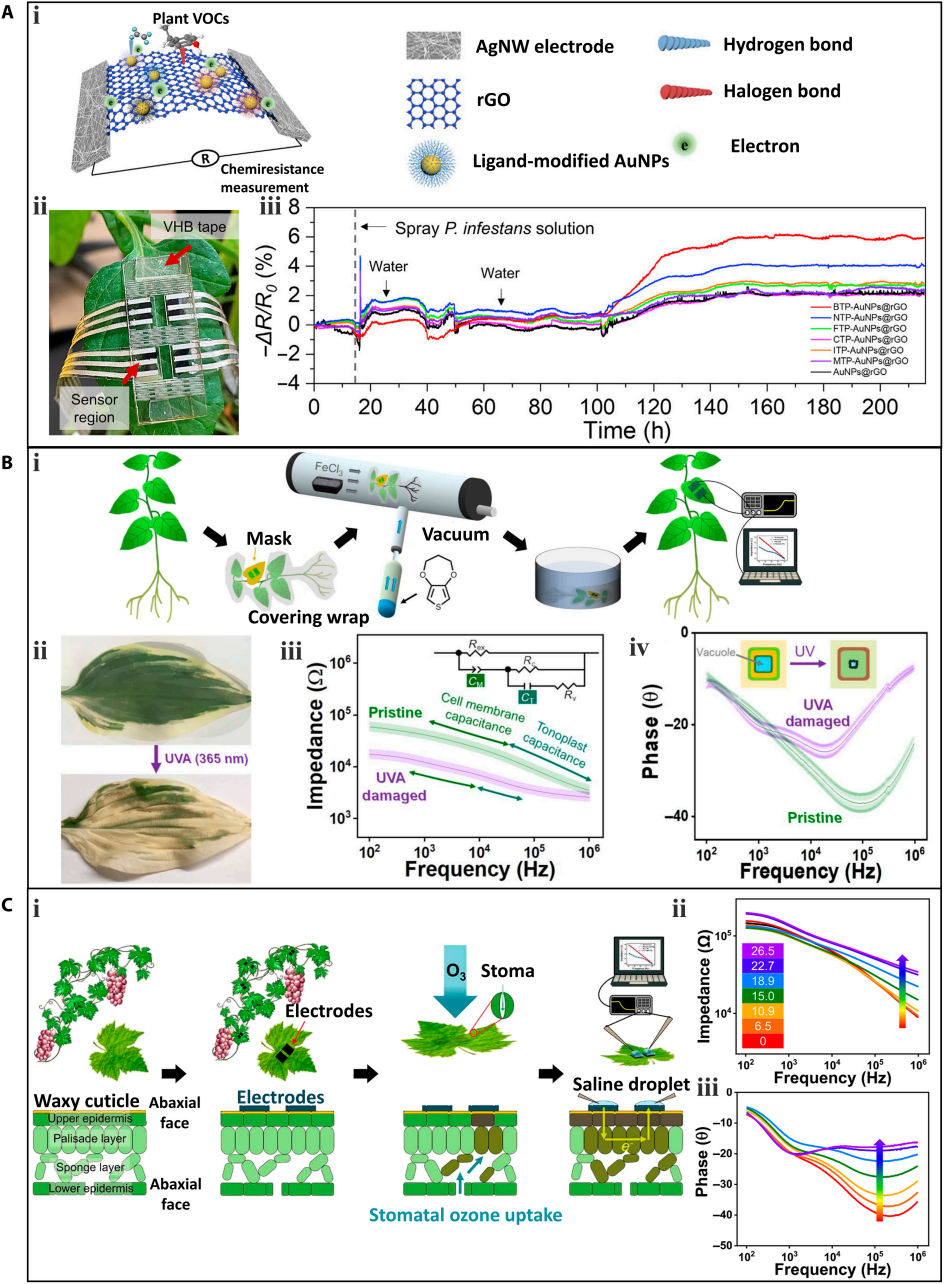
Wearable sensors for the measurement of plant stress response. (A) A sensor for detecting VOCs emitted by plants under pathogen infection: (i) a schematic of the sensor with soft AgNW electrodes and graphene-based sensing materials, (ii) a photograph of wearable sensor attached on the tomato leaf, and (iii) real-time response of the sensor array to inoculation of infectious *Plasmodium*. Reproduced with permission. [80] Copyright 2021, Elsevier. (B) A sensor for detecting impedance spectroscopy of plant leaves under UVA irradiation: (i) schematics of sensor fabrication and measurement, (ii) photographs of a pristine and UVA-irradiated *Hosta* leaf, and (iii) frequency-dependent impedance and (iv) phase response of a pristine (green) and UVA-irradiated (purple) *Hosta* leaf. Reproduced under the terms of the CC-BY 4.0 Creative Commons Attribution License. [82] Copyright 2019, the Authors, published by AAAS. (C) A sensor for detecting impedance spectroscopy of plant leaves under ozone oxidation: (i) schematics of sensor fabrication and measurement, and (ii) impedance and (iii) phase responses under varying ozone concentrations. Reproduced under the terms of the CC-BY 4.0 Creative Commons Attribution License. [44] Copyright 2020, the Authors, published by AAAS. VHB tape, "Very High Bond" tape.

*Phytophthora infestans* (*P. infestans*) is responsible for causing plant late blight, a destructive disease that affects various plants, including tomato and potato [79]. The infected plants usually emit volatile organic compounds (VOC) gases, such as aldehydes, during the early stage. Li et al. [80] demonstrated the use of a gas sensor array attached to leaves for the early-stage identification of late blight caused by *P. infestans*. The sensing mechanism of the sensor is shown in Fig. 6Ai. The sensor array consists of gold nanoparticles (AuNPs) decorated reduced GO (rGO) and silver nanowire (AgNW) acting as the sensing layer and electrode, respectively. The sensing layer can form reversible interactions with plant VOCs by hydrogen or halogen bonds, resulting in a resistance increase of the sensor. The sensor array was attached to a tomato leaf using double-sided tape (Fig. 6Aii). The real-time response curves of the gas sensor array are shown in Fig. 6Aiii. After 15 h of stable sensor response, the whole plant was sprayed with a suspension of infectious *P. infestans* sporangia. Small fluctuations in the signal were observed during the first 35 h. A marked increase was observed at 100 h, indicating the emission of characteristic VOC gases induced by the propagation of *P*. *infestans* infection. Notably, 2 watering events at 25 and 35 h induced negligible signal interference. After 115 h, the signals gradually stabilized, indicating that the tomato leaf was completely infected by *P. infestans.* It is worth mentioning that at 115 h, typical symptoms of late blight, including water-soaked lesions and circular gray spots, started to become visible on the leaves. The results confirm the potential of the sensor array for the identification of VOCs during the early stage of *P. infestans* infection.

Ultraviolet A (UVA) radiation can cause green leaves to turn yellow and even fall off, leading to marked DNA damage and changes in biomass accumulation and resource allocation in plants [81]. The impedance spectroscopy of plant tissues will change under UVA stress, which can be used as an indicator. To detect UVA-induced stress, Kim et al. [82] developed a biocompatible conductive polymer electrode that can be directly printed onto living plants for conformal and long-term health monitoring, as shown in Fig. 6Bi. After being exposed to UVA radiation for 4 h (equivalent to 9.5-d-daylight exposure), a pristine *Hosta* leaf turned pale yellow due to a decrease in chlorophyll content (Fig. 6Bii). The frequency-dependent impedance and phase of *Hosta* leaves measured by the printed conductive polymer electrodes were markedly different for the healthy pristine and damaged leaf (Fig. 6 Biii to iv). The equivalent circuit used to simulate the bioimpedance response of the host plant leaf is shown in Fig. 6Biii and consists of 2 capacitive tissue components (C_M_ and C_T_). Under UVA irradiation, the values of C_M_ and C_T_ increased by 370% and 80%, respectively.

In addition to UVA irradiation, the conductive polymer electrodes can also detect the impedance change induced by ozone oxidation [44]. Long-term exposure to ground-level ozone, which is produced by the reaction of nitrate in the topsoil and some air pollutants with oxygen, can cause irreversible oxidation damage to plants [83,84]. Ozone disrupts the normal redox process of plant cells, and high ozone concentrations can inhibit plant's ability to absorb carbon dioxide. The conductive polymer electrodes were deposited on freshly cut grape leaves and then exposed to different concentrations of ozone (Fig. 6Ci). The oxidative damage caused by ozone exposure exhibited a unique change in the impedance and phase signals of leaf tissue, which were monitored by the wearable sensors (Fig. 6 Cii to iii). In the high-frequency region (10^4^ to 10^6^ Hz), both the impedance and phase increased with the ozone exposure concentration.

## Wearable Sensors for Plant Environment Monitoring

The environment is 1 of 2 crucial factors determining plant phenotypes, making the monitoring of the environment an essential aspect of plant phenotyping. Optical methods, including machine vision [85], spectroscopy [86], and aerial vehicle [87], are conventional techniques for monitoring the environment around plants, providing large area coverage. However, these methods are limited for detecting the microenvironment that directly affects plant growth. In contrast, wearable sensors with contact measurement mode can closely adhere to the surface of plants, sensing real-time changes in the microenvironment. This section reviews the progress of wearable sensors for monitoring the environment, including air temperature, air moisture, light, pesticides, and toxic gas, as summarized in Table 2. Notably, multimodal sensors are typically integrated to simultaneously monitor these environmental factors.

**Table 2.** Wearable sensors for the measurement of environment.

Environment	Sensor	Plant	Ref.
Air temperature	A Au-based integrated wearable device	*Scindapsus aureus*	[50]
	A Au-based multifunctional stretchable sensor	Corn	[45]
	A multimodal flexible sensor based on SnO_2_ and SWCNTs	*P. macrocarpa*	[47]
Air moisture	An integrated wearable device	*Scindapsus aureus*	[50]
	A multimodal flexible sensor	*P. macrocarpa*	[47]
Light	A multifunctional stretchable sensor	Corn	[45]
	A multimodal flexible sensor	*P. macrocarpa*	[47]
Pesticide	A LIG-based wearable sensor	Spinach	[92]
Toxic gas	A device based on SWCNT channels and graphite electrodes	\	[95]
	A sprayed NO_2_ gas sensor array	Lucky bamboo	[43]

### Air temperature

Air temperature can have a marked impact on photosynthesis, which is a vital process for producing energy and sugar for plant growth. Inadequate or excessive temperature levels can hinder the healthy development of plants.

**Fig. 7.**
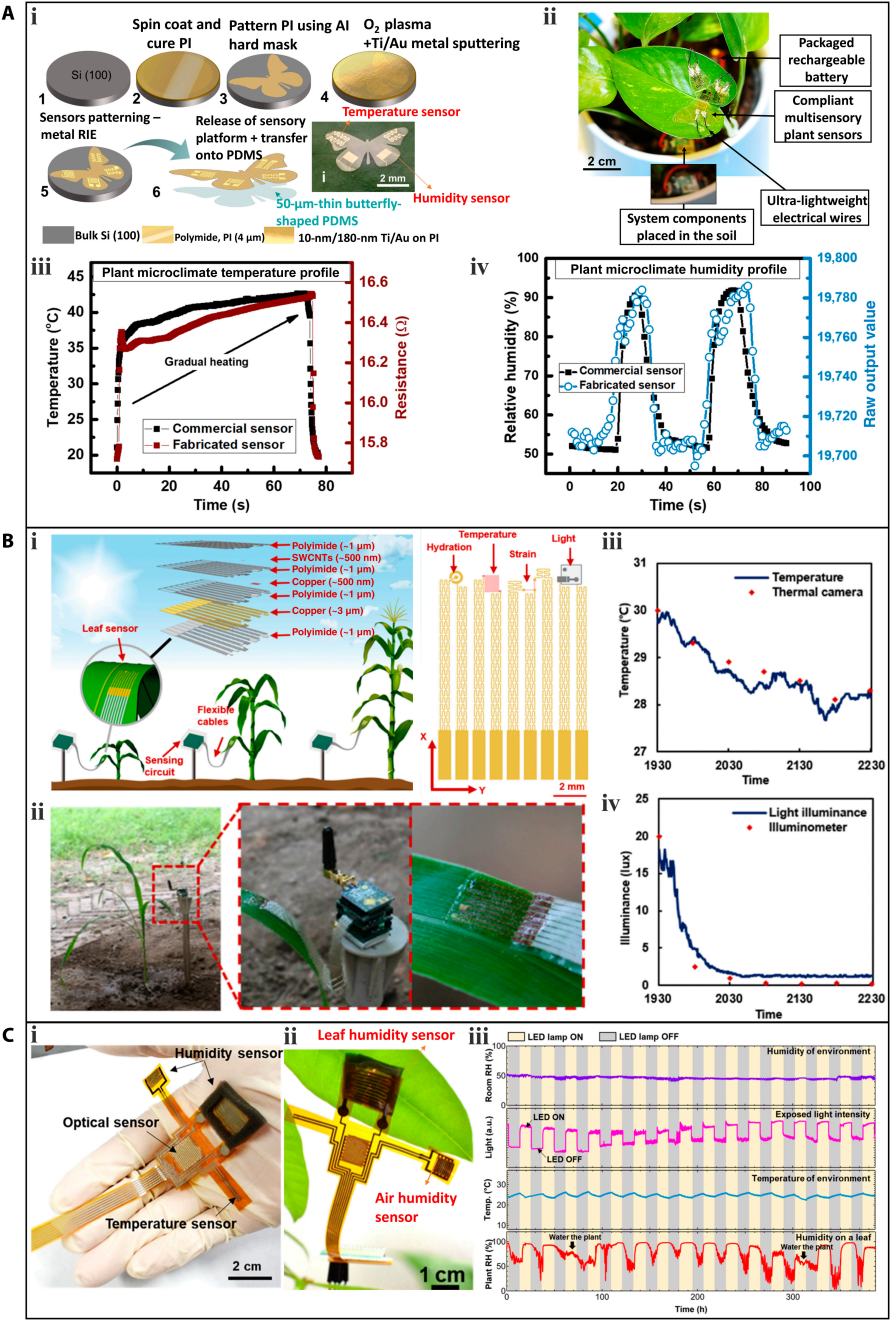
Wearable sensors for the measurement of air temperature, air moisture, and light. (A) A wearable device with integrated temperature and humidity sensors: (i) the fabrication process flow chart of the flexible multisensory platform, (ii) a photograph of the integrated wearable sensor system, (iii) real-time response of the temperature sensor when exposed to local variation in the temperature profile, and (iv) real-time response of the humidity sensor in response to variation in the humidity levels. Adapted under the terms of the CC-BY 4.0 Creative Commons Attribution License. [50] Copyright 2018, the Authors, published by Springer Nature. (B) A lightweight stretchable sensor with multiple sensing elements: (i) an exploded view (left) and top view (right) of the multifunctional leaf sensor, (ii) photographs of the multifunctional leaf sensor mounted on a corn leaf, and measured results of (iii) ambient temperature and (iv) light intensity. Reproduced under the terms of the CC-BY 4.0 Creative Commons Attribution License. [45] Copyright 2019, the Authors, published by ACS. (C) A multimodal flexible sensor system: (i) a photograph of the device, (ii) a photograph of the working scenario, and (iii) monitoring results of the multimodal sensor for over 350 h. Leaf humidity, room temperature, light, and room humidity results were displayed from the bottom to the top. Adapted with permission. [47] Copyright 2020, ACS. LED, light-emitting diode.

Nassar et al. [50] developed a wearable device that integrates temperature and humidity sensors, which can be deployed on plant surfaces. The flexible sensory platform was fabricated using traditional Si-based microfabrication technology. As shown in Fig. 7Ai, electrodes made of ultralight butterfly-shaped PI were sputtered with Au. Among these, the serpentine Au pattern acted as the temperature sensor, as the resistance of Au increases with temperature (0.032 Ω/°C). The sensory platform was placed on the surface of *Scindapsus aureus* leaves and connected to data acquisition and transmission circuits using ultralight electrical wires and silver epoxy (Fig. 7Aii). The developed flexible sensory platform monitored the real-time air temperature around the plant. To confirm the sensing performance of the temperature sensor, the data generated by the system was compared with the data collected by a commercial sensor (Fig. 7Aiii). As the temperature increased (read by the commercial temperature sensor), the resistance of the developed temperature sensor increased synchronously. The results demonstrated the good reliability of the fabricated sensor.

Multifunctionality is a key advantage of plant wearable sensors. A lightweight and stretchable sensor, capable of monitoring multiple plant phenotypes (elongation and hydration) and environmental factors (air temperature and light), is shown in Fig. 7Bi [45]. The entire sensor weighs only 17 mg and has a large stretchability of 120%, facilitated by a self-similar serpentine design. These features minimize interference with the growth of the host leaf. The temperature sensing element utilizes a Cu layer with a meander pattern. The sensor was installed on a corn leaf outdoors to monitor real-time air temperature (Fig. 7Bii). The recorded temperature data was consistent with data obtained using a thermal imaging camera (Fig. 7Biii).

Another multimodal flexible sensor system, which consists of a temperature sensor, an optical sensor, and 2 humidity sensors, was developed for measuring the environment (Fig. 7Ci) [47]. All 3 types of sensors were fabricated on a flexible PI substrate that was 50 μm thick. For the temperature sensor, Ag electrodes were screen-printed on the PI substrate. SnO_2_ nanoparticles and single-walled carbon nanotubes (SWCNTs) were then deposited onto the Ag electrodes as the temperature sensing element. Finally, a passivation layer was coated onto the temperature sensor to prevent the effects of humidity and scratches. The integrated sensor system weighed approximately 0.3 g and was attached to the lower epidermis of a leaf of *P*. *macrocarpa* (an evergreen indoor plant) for real-time monitoring (Fig. 7Cii). The third row of Fig. 7Ciii shows the air temperature measurement for almost 16 d, which revealed a steady temperature of approximately 25 °C.

### Air moisture

Air humidity is a crucial factor that affects stomatal opening and closing, thereby regulating the plant's transpiration rate, which controls water absorption and mineral nutrition transport. The moisture in the air also has a direct impact on plant health. If the humidity is too low, plant leaves tend to wilt and detach to conserve water, impeding plant growth. Conversely, if the humidity is too high, plants are vulnerable to insect infestations as well as foliar and root diseases.

The ultralight butterfly-shaped flexible multisensory platform shown in Fig. 7Ai also includes a humidity sensor with an interdigital shape. In this case, PI serves as the humidity sensing element, and its capacitance increases with humidity, displaying a high sensitivity of 1.6/% RH. When installed on a plant leaf for real-time environmental monitoring (Fig. 7Aii), the data collected from the fabricated humidity sensor over 2 periods was consistent with that obtained from a commercial humidity sensor (Fig. 7Aiv).

The multimodal flexible sensor system depicted in Fig. 7 Ci had 2 humidity sensors, both of which were fabricated by generating interdigital LIG electrodes on the PI substrate through laser scanning. The humidity sensing element for both sensors was ZnIn_2_S_4_ nanosheets deposited on the LIG electrodes. One sensor was exposed to the atmosphere for the measurement of air humidity (room humidity), while the other was attached directly to the lower epidermis of a *P. macrocarpa* leaf for the measurement of leaf humidity. During the plant's growth, the air humidity was maintained at a constant level, and the light was periodically switched on and off (second row of Fig. 7Ciii). The data recorded by the smaller humidity sensor (first row of Fig. 7Ciii) confirmed the constant level of air humidity, while the data measured by the larger humidity sensor (fourth row of Fig. 7Ciii) indicated that leaf humidity rapidly increased when the light was on, and stomata opened for photosynthesis. Conversely, the leaf humidity decreased when the light source was turned off.

### Light

Light is one of the most important environmental factors for plants. On one hand, light is indispensable for photosynthesis, while on the other hand, excessive light can cause physical damage to plants, such as leaf burning [88]. Therefore, monitoring the light intensity in the environment is crucial.

In the previously mentioned stretchable multimodal sensor (Fig. 7Bi), a silicon-based phototransistor was used for light sensing. To improve flexibility and reduce weight, the phototransistor was mechanically polished to a thickness of 20 μm. During real-time monitoring of a corn leaf outdoors (Fig. 7Bii), the phototransistor detected the light attenuation during sunset, and the measurement result was consistent with that measured by a commercial illuminometer (Fig. 7Biv).

The multimodal flexible sensor system illustrated in Fig. 7Ci featured an optical sensor, which was fabricated by screen-printing Ag electrodes onto the PI substrate and depositing ZnIn_2_S_4_ nanosheets onto the Ag electrodes as the light sensing element. The optical sensor exhibited a fast response time of approximately 4 ms and could detect light illumination at a frequency of 50 Hz. To simulate day and night, an artificial light source (18 W) was automatically switched on and off every 12 h, and the switching was accurately detected by the wearable sensor (Fig. 7Ciii).

### Pesticide

Pesticides are widely used in agriculture to protect plants from insect pests [89]. However, they can also leave behind residues that can affect plant phenotypes. Current methods for detecting pesticide residues include mass spectrometry [90], high-performance liquid chromatography [90], and gas chromatography [91]. However, these methods require expensive equipment and are not suitable for in-situ detection.

**Fig. 8.**
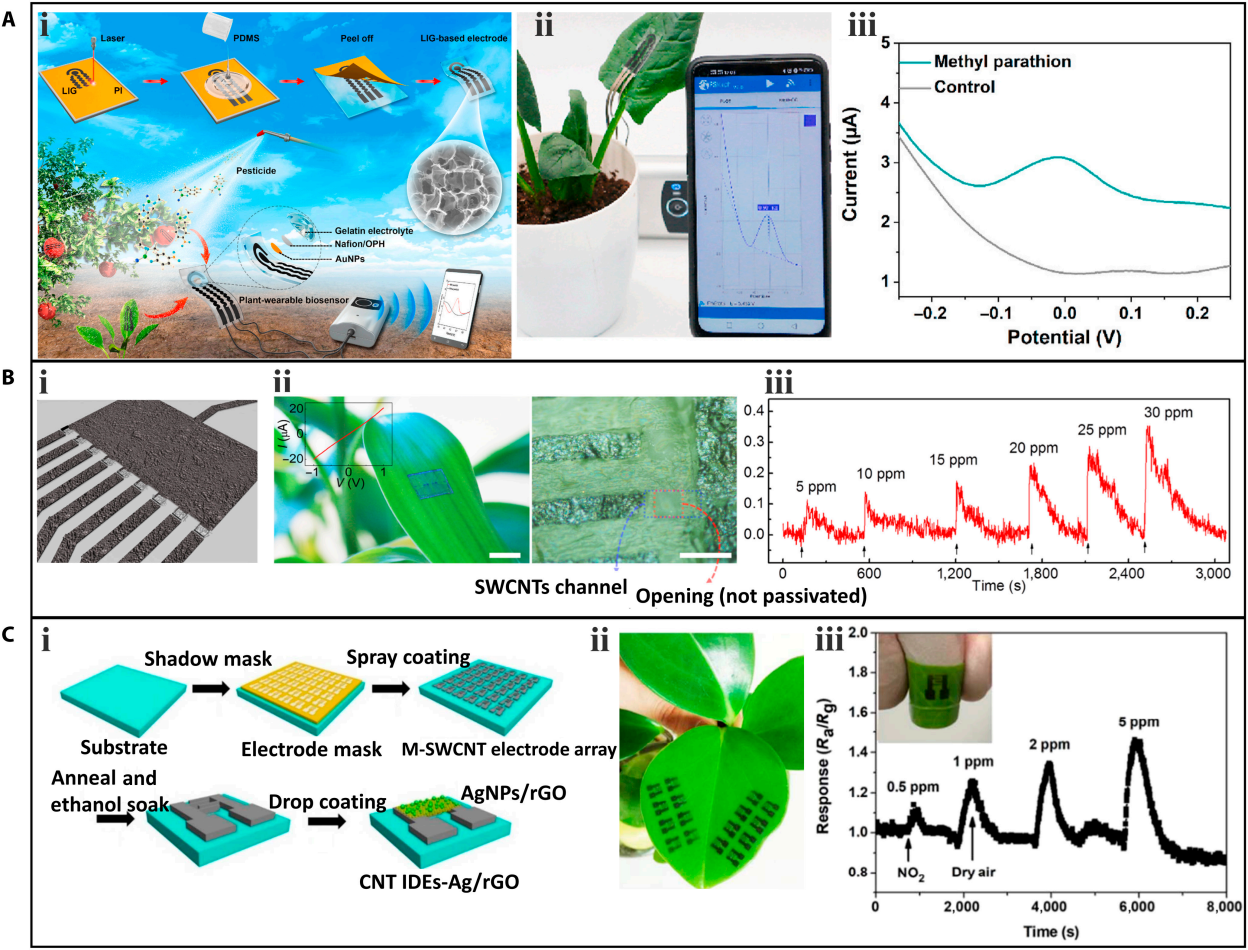
Wearable sensors for the measurement of pesticide and toxic gas. (A) A wearable sensor for monitoring organophosphorus pesticides: (i) schematic of the manufacturing process of the wearable sensor, (ii) a photograph of the sensor attached to a spinach plant, and (iii) square wave voltammetry curves of sample sprayed with methyl parathion and control sample without methyl parathion. Reproduced with permission. [92] Copyright 2020, Elsevier. (B) A DMMP gas sensor array: (i) a schematic diagram of the DMMP gas sensor array, (ii) photographs of the DMMP gas sensors transferred onto the surface of a live leaf, and (iii) real-time response to varying concentrations of DMMP. Reproduced with permission. [95] Copyright 2014, ACS. (C) A NO_2_ gas sensor array: (i) schematics of the manufacturing process of the NO_2_ gas sensor array, (ii) a photograph of the gas sensor array fabricated on a living plant leaf, and (iii) dynamic responses of the gas sensor array to NO_2_ gas. Adapted with permission. [43] Copyright 2018, ACS. OPH, organophosphorus hydrolase M-SWCNT, metallic single-walled carbon nanotube; CNT, carbon nanotube.

Wearable sensors have been utilized to detect pesticide residues on plants. Zhao et al. [92] developed a wearable sensor that can be directly attached to the plant surface for in-situ detection of organophosphorus pesticides. The fabrication process of the sensor is illustrated in Fig. 8Ai. Serpentine 3-electrode LIG was synthesized on a PI film and transferred to PDMS. The prepared LIG electrodes on the PDMS substrate had good flexibility and stretchability, which can well adapt to the irregular surface of plants. Then, the LIG-based electrodes were modified with organophosphorus hydrolase and AuNPs to enhance the electrochemical performance. The sensor was affixed to the surface of a spinach leaf for in-situ detection (Fig. 8Aii). When methyl parathion solution was sprayed onto the leaf surface, the sensor acquired real-time information on pesticide residues and displayed it on a smartphone (Fig. 8Aiii). A clear peak of p-nitrophenol was observed when the methyl parathion was present compared to the control experiment.

### Toxic gas

Toxic gases in the environment, even in small amounts, can cause irreversible damage to plants [93]. Current detection of these gases mainly relies on gas chromatography [94], which is a costly and time-consuming process. Furthermore, it can be challenging to collect gas samples in the field where airflow disturbance frequently occurs. Wearable sensors can provide a solution to these challenges by performing in-situ measurements of toxic gases.

A gas sensor array based on SWCNT channels and graphite electrodes was used to detect the simulants of sarin nerve agent, dimethyl methylphosphonate (DMMP), which can interfere with the photosynthetic process of plants [95]. The gas sensor array consisted of 9 field-effect sensors (Fig. 8Bi). The resistance of the SWCNT channels with openings around them could be modulated by the molecules adsorbed on the surface of the SWCNT donating or withdrawing electrons. Additionally, the gas sensor array exhibited good adhesion and could be easily transferred to planar and nonplanar surfaces. As shown in Fig. 8Bii, the array was transferred to the leaf surface of a lucky bamboo to sense DMMP gas. When DMMP gas was present, the sensor responded within 5 s, and the response intensity increased with the DMMP concentration (Fig. 8Biii).

Another toxic gas, nitrogen dioxide (NO_2_), can cause plant wilt and leaf yellowing [96]. A sprayed gas sensor array was developed using metallic SWCNTs as the conductive electrode and AgNPs/rGO as the sensing element (Fig. 8Ci) [43]. The sensor was directly sprayed onto the leaves of living plants for in-situ detection of NO_2_ (Fig. 8Cii). The obtained gas dynamic response is shown in Fig. 8Ciii. When NO_2_ was exerted onto the plant, the sensor's resistance rapidly increased, and this response was reversible after NO_2_ was replaced by dry air. As the concentration of NO_2_ increased, the response of the sensor also increased. The limit of detection is as low as 0.5 ppm. The sprayed sensor has better detection performance compared to conventional metal electrode-based sensors [97], demonstrating its great potential in the in-situ detection of NO_2_ around plants.

## Challenges and Perspectives

**Fig. 9.**
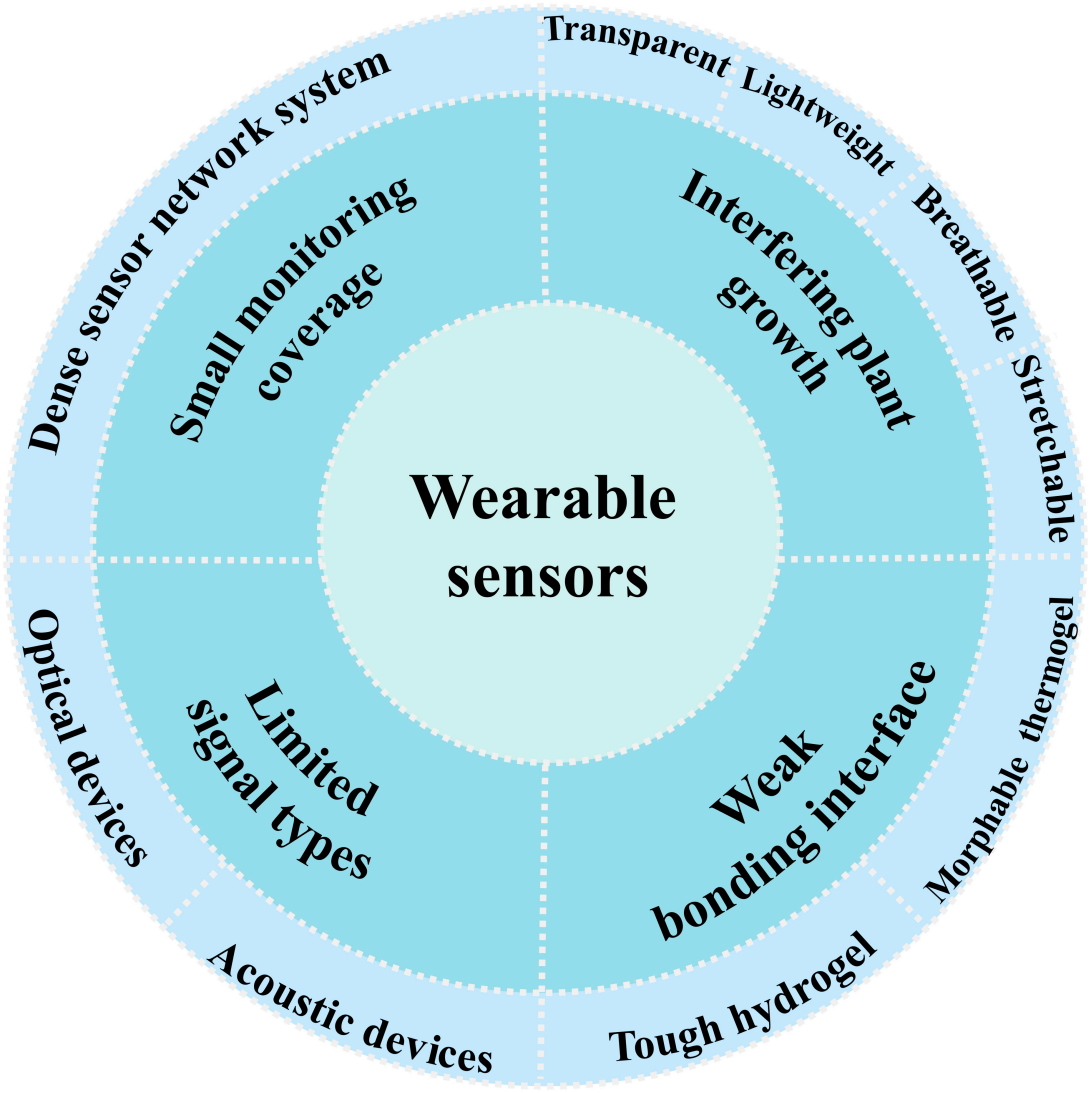
Schematic of the present challenges and future directions of wearable sensors for plant phenotyping.

Wearable sensors hold great promise for plant phenotyping due to their high spatial resolution [98], multifunctionality [99], and minimal invasiveness [100]. A few commercial plant wearable sensors are already commercially available. For example, AgriHouse Inc. has released a plant wearable sensor named “Leaf Sensor” for the measurement of plant water level. However, several challenges remain in the transition from concept demonstration to large-scale application [101], including interference with plant growth, weak bonding interface, limited signal type, and small monitoring coverage. We have summarized these challenges and provided potential solutions (Fig. 9):

1. Interfering plant growth. While wearable sensors can be less invasive than some other sampling methods, they can still interfere with plant growth. For example, the weight of the sensor can create pressure on the plant, and the sensor may not grow synchronously with the host plant. Additionally, the sensor can cover stomata, hindering gas exchange, and may reduce light absorption due to its opaqueness. Therefore, to minimize interference plant wearable sensors should be lightweight, soft, stretchable, breathable, and transparent, which can be satisfied from material selection and structural design [102].

2. Weak bonding interface. To achieve real-time measurements, the wearable sensor must remain attached to the host plant continuously. Thus, a strong bonding interface is required between the sensor and the plant. However, the plant's epidermis is typically irregular and uneven due to the presence of microstructures such as stomata, mastoid, and villi, which provide limited bonding sites for sensors with smooth surfaces. Previous research has used clamps to fix wearable sensors, but the mechanical pressure can interfere with plant growth [103]. Advanced technology, as demonstrated in Fig. 5C, utilizes a morphable thermogel to compensate for the morphological mismatch between the plant and the sensor. More solutions can be inspired by tough hydrogels to address this challenge [104,105].

3. Limited signal types. Currently, wearable sensors are electronic devices that convert plant phenotype and environmental information into electrical signals. As a result, only a limited signal type can be collected. For example, current wearable electronic sensors have not been able to measure nitrogen content, a critical phenotype indicator. To obtain more signal types, other devices such as optical and acoustic devices can be integrated into wearable sensors [106].

4. Small monitoring coverage. While wearable sensors have high spatial resolution, the information they acquire is local. Currently, only a limited number of wearable sensors are attached to a leaf or stem of a plant, which cannot monitor the overall phenotype and environmental information of the host plant, let alone the information of other plants in the same field. To expand the monitoring coverage, numerous wearable sensors are expected to be distributed over the target field to build a dense sensor network system. This requires wearable sensors to be produced at a large scale and low cost [68].

## Conclusion

In this review, we have provided a comprehensive overview of the progress made in the development of wearable sensors for monitoring plant phenotypes (including elongation, leaf temperature, hydration, bioelectric potential, and stress response) and environment (including air temperature, humidity, light, pesticide, and toxic gas). Compared to traditional phenotyping technologies based on optical imaging, wearable sensors have unique advantages, such as high spatial resolution, the ability to easily uncover the impact of environmental factors on phenotypes, and high accuracy in fields, which demonstrate their great potential in plant phenotyping. Although challenges exist, such as interfering with plant growth, weak bonding interfaces, limited signal types, and small monitoring coverage, we have proposed possible solutions. With the continued progress and improvement of wearable sensors, they will markedly accelerate plant phenotyping.

## Data Availability

This review paper does not contain research data to be shared.
